# Supplementary material for: High-resolution European daily soil moisture derived with machine learning (2003–2020)
Source: Sci Data. 2022 Nov 14;9:701. doi: 10.1038/s41597-022-01785-6 (PMC9663700; doi:10.1038/s41597-022-01785-6)
Supplement: Supplementary file 1 — Supplementary Information [file 41597_2022_1785_MOESM1_ESM.pdf]

High-resolution European daily soil moisture derived with machine learning (2003-2020)

Sungmin O[1,*], Rene Orth[2], Ulrich Weber[2], and Seon Ki Park[1,3,4]

[1]Department of Climate Energy System Engineering, Ewha Womans University, Korea
[2]Department of Biogeochemical Integration, Max Planck Institute for Biogeochemistry, Jena, Germany
[3]Center for Climate/Environment Change Prediction Research, Ewha Womans University, Korea
[4]Severe Storm Research Center, Ewha Womans University, Korea
[*]corresponding author(s): Sungmin O (sungmino@ewha.ac.kr), Seon Ki Park (spark@ewha.ac.kr)

Contents

Supplementary Figure 1 2

Supplementary Figure 2 3

Supplementary Figure 3 4

Supplementary Figure 4 5

Supplementary Figure 5 6

Supplementary Figure 6 7

Supplementary Figure 7 8

Supplementary Figure 8 9

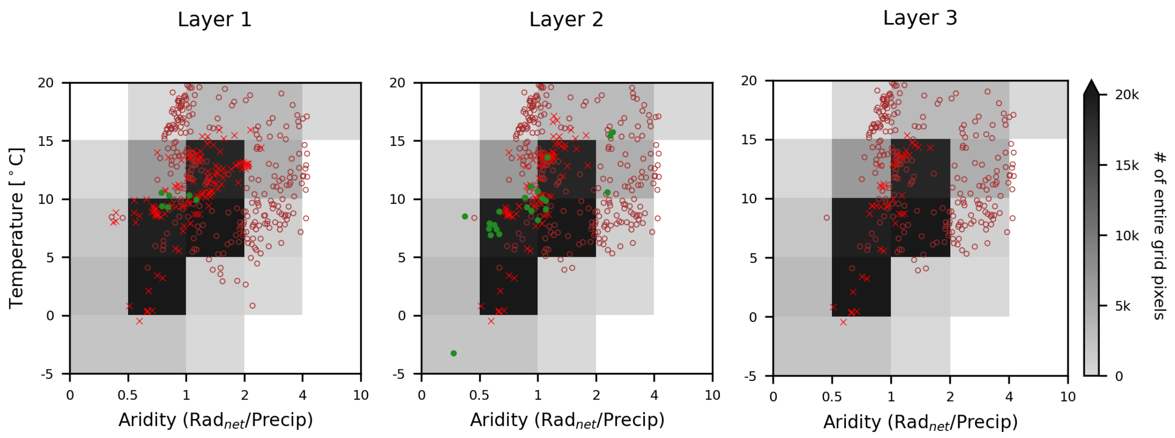

Figure S1. Same as Fig. 2(c) in the main text, but also for the second and third layers. Green markers indicate the independent in-situ data from COSMOS-Europe used for the final data validation; those are not available for Layer 3.

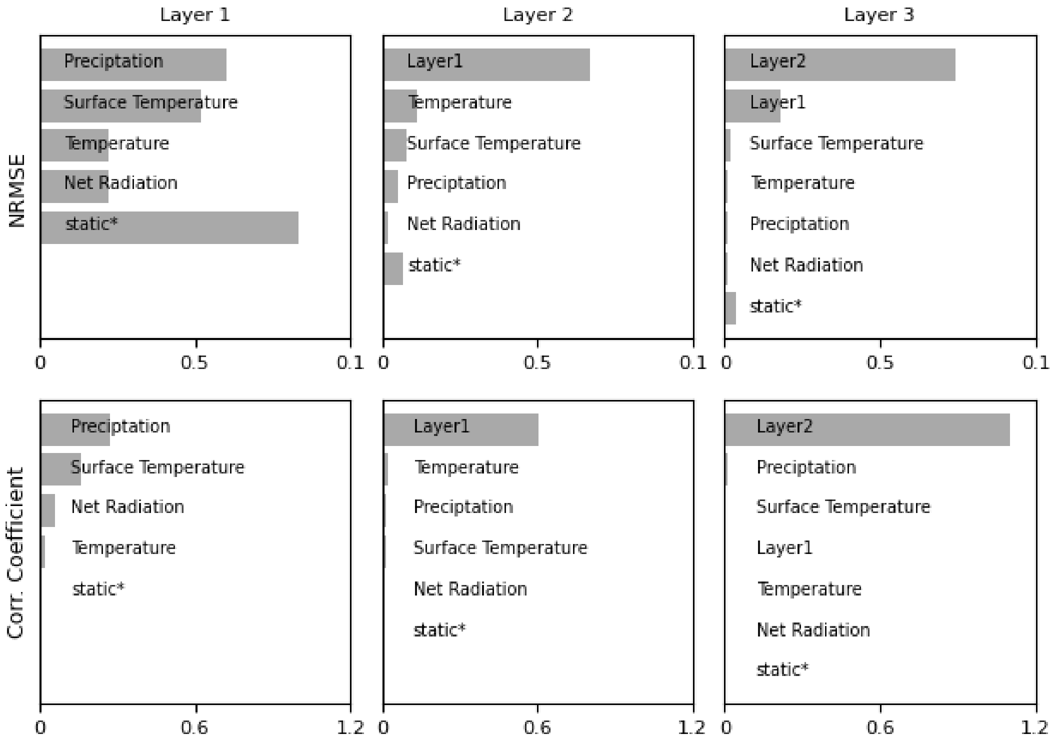

Figure S2. The relative importance of input variables. We randomly shuffle the time series of each input variable and compare the respective decreases in model performance. An input variable is important if shuffling increases the model error; normalised root-mean-square error (NRMSE) and correlation coefficient are used for the statistical analysis. For the static features, we permute all variables together across space.

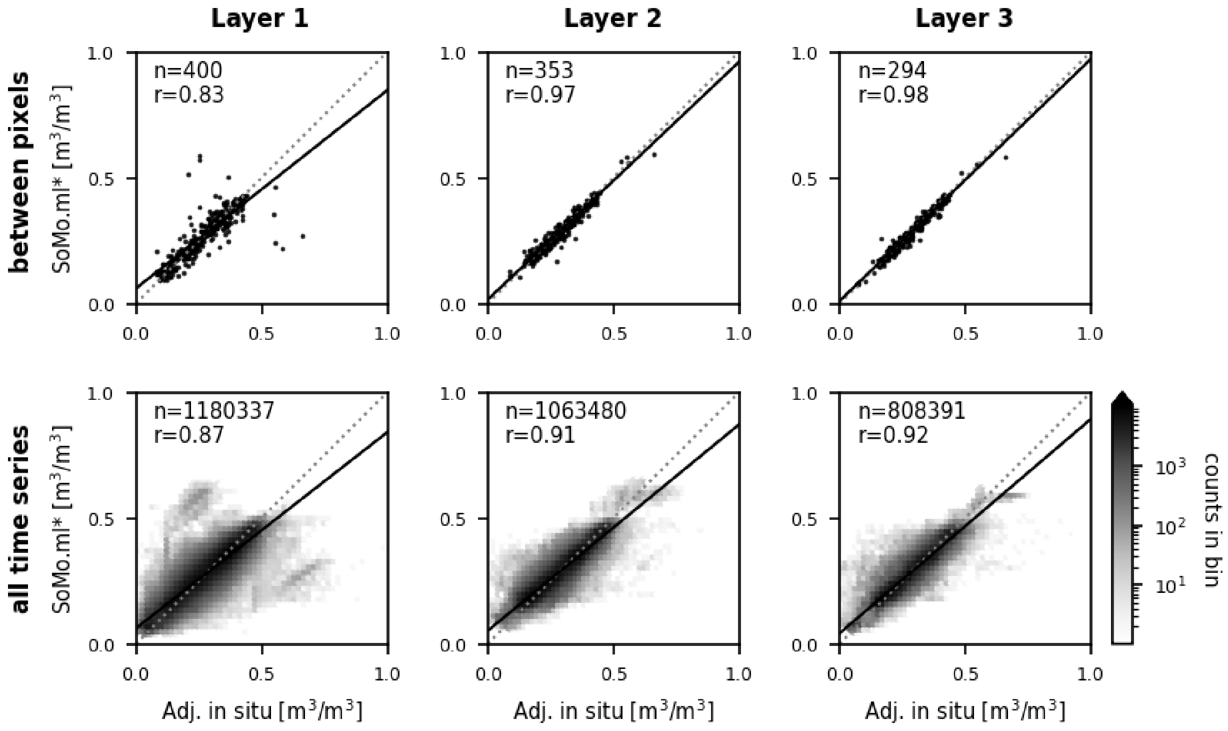

Figure S3. Same as Fig. 4 in the main text, but this time we perform the five-fold cross-validation. The entire training data is spatially split into five subsets.

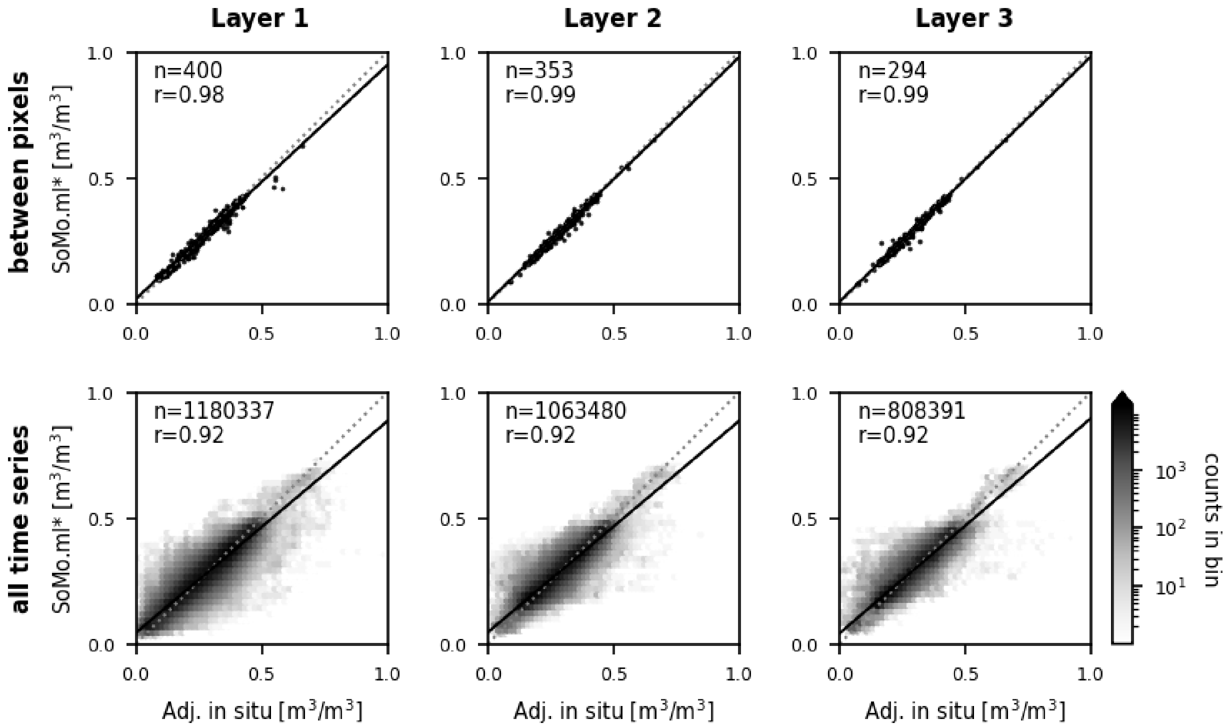

Figure S4. Same as Fig. S3, but for the five-fold cross-validation over time. The entire training data is temporally split into five subsets.

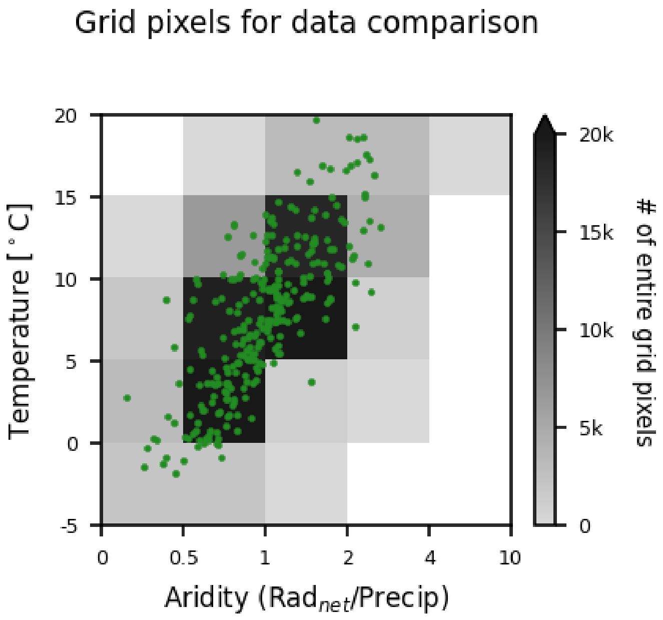

Figure S5. We select pixels from every 20 x 20 pixel segment over the entire domain to intercompare the soil moisture datasets across diverse climate regimes.

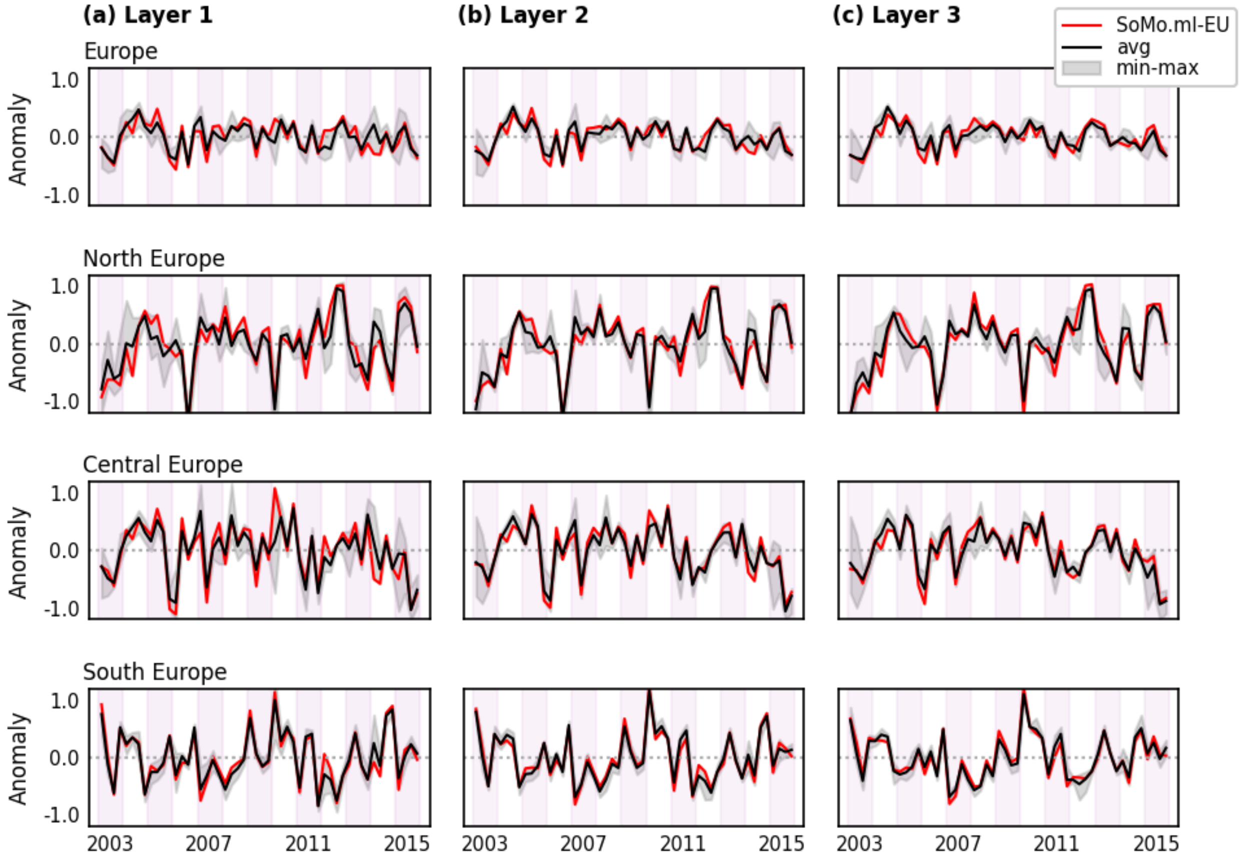

Figure S6. The normalised seasonal soil moisture anomalies over the period 2003-2015 over the entire European domain, Northern, Central, and Southern Europe (top to bottom). Black lines show the average of all the datasets considered for each layer (See Fig. 6 in the main text) and red lines show SoMo.ml-EU. The shaded areas indicate the range of anomalies (maximum and minimum) across the considered reference datasets.

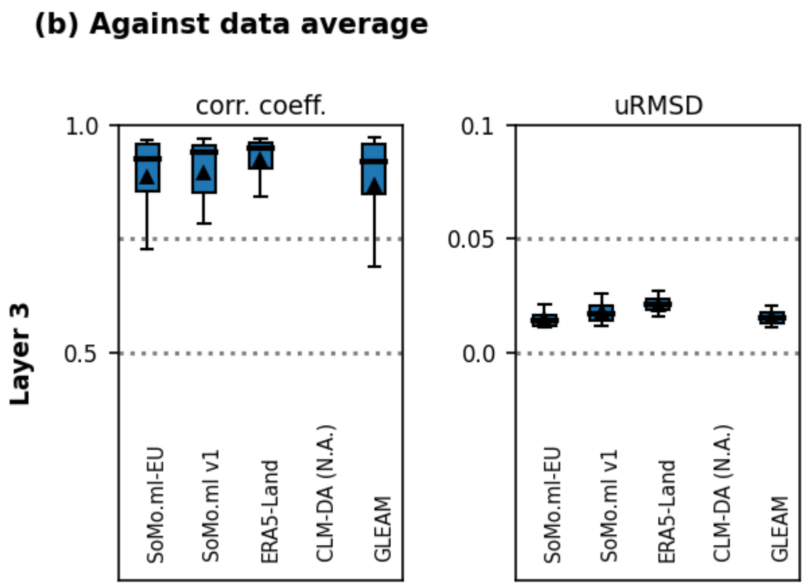

Figure S7. Same as Fig. 5(b) in the main text, but for Layer 3.

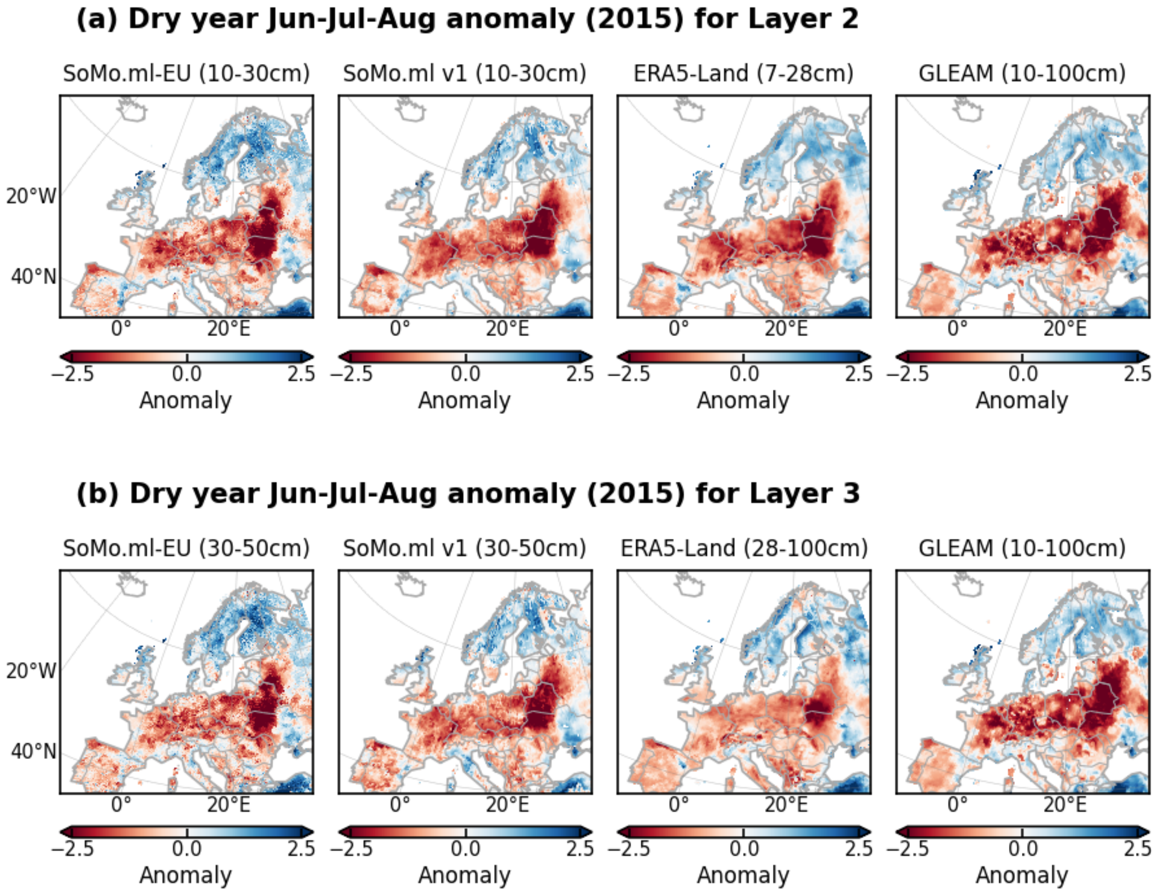

Figure S8. Same as Fig. 6(b) in the main text, but for Layer 2 and Layer 3. CLM-DA is not available for the deeper layers.
